# Anti-HIV potency of T-cell responses elicited by dendritic cell therapeutic vaccination

**DOI:** 10.1371/journal.ppat.1008011

**Published:** 2019-09-09

**Authors:** Mathieu Surenaud, Monica Montes, Cecilia S. Lindestam Arlehamn, Alessandro Sette, Jacques Banchereau, Karolina Palucka, Jean-Daniel Lelièvre, Christine Lacabaratz, Yves Lévy

**Affiliations:** 1 Vaccine Research Institute, INSERM U955—Université Paris-Est Créteil, Créteil, France; 2 Baylor Institute for Immunology Research, Center for Human Vaccines, Dallas TX, United States of America; 3 La Jolla Institute for Immunology, Department of Vaccine Discovery, La Jolla, California, United States of America; 4 University of California San Diego, Department of Medicine, La Jolla, California, United States of America; 5 Assistance Publique-Hôpitaux de Paris, Groupe Henri-Mondor Albert-Chenevier, Service d’Immunologie Clinique, Créteil, France; Emory University, UNITED STATES

## Abstract

Identification and characterization of CD8^+^ and CD4^+^ T-cell epitopes elicited by HIV therapeutic vaccination is key for elucidating the nature of protective cellular responses and mechanism of the immune evasion of HIV. Here, we report the characterization of HIV-specific T-cell responses in cART (combination antiretroviral therapy) treated HIV-1 infected patients after vaccination with *ex vivo*-generated IFNα Dendritic Cells (DCs) loaded with LIPO-5 (HIV-1 Nef 66–97, Nef 116–145, Gag 17–35, Gag 253–284 and Pol 325–355 lipopeptides). Vaccination induced and/or expanded HIV-specific CD8^+^ T cells producing IFNγ, perforin, granzyme A and granzyme B, and also CD4^+^ T cells secreting IFNγ, IL-2 and IL-13. These responses were directed against dominant and subdominant epitopes representing all vaccine regions; Gag, Pol and Nef. Interestingly, IL-2 and IL-13 produced by CD4^+^ T cells were negatively correlated with the peak of viral replication following analytic treatment interruption (ATI). Epitope mapping confirmed that vaccination elicited responses against predicted T-cell epitopes, but also allowed to identify a set of 8 new HIV-1 HLA-DR-restricted CD4^+^ T-cell epitopes. These results may help to better design future DC therapeutic vaccines and underscore the role of vaccine-elicited CD4^+^ T-cell responses to achieve control of HIV replication.

## Author summary

Improvement of therapeutic vaccine strategies in the perspective of HIV cure is warranted and one of the determinant factors for elucidating the nature of protective cellular responses is the identification and characterization of CD8^+^ and CD4^+^ T-cell epitopes elicited by the vaccine. However, fine characterization of vaccine response at the level of individual peptides contained in therapeutic vaccines is lacking. Here, we report in-depth characterization of HIV-specific T-cell responses observed in antiretroviral therapy treated HIV-1 infected patients after vaccination with autologous *ex vivo*-generated IFNα DCs loaded with 5 long HIV peptides coupled to a lipid tail. Our study shows that dominant and subdominant epitopes from Gag, Pol, and Nef vaccine regions are targeted by HIV-specific CD8^+^ T and CD4^+^ T cells and that vaccine-elicited HIV-specific CD4^+^ T cells producing IL-2 and IL-13 are significantly correlated with a better viral control following treatment interruption. These results underscore the role of vaccine-elicited CD4^+^ T-cell responses to achieve control of HIV replication and thus may inform the design of better therapeutic vaccines.

## Introduction

Globally, an estimated 36.9 million people were living with Human Immunodeficiency Virus (HIV) in 2017, among them 59% were accessing antiretroviral therapy [1]. Introduction of combination antiretroviral therapy (cART) since the late 1990’s decreased HIV-related morbidity and mortality [2] and reduced the risk of HIV transmission [3]. However, cART requires a life-long adherence to treatment, has potential long-term side-effects [4], and poor adherence can result in the development of resistance [5]. HIV infection has also been shown to result in more prevalent age-associated non-communicable comorbidities like peripheral arterial, cardiovascular disease, and impaired renal function as compared to HIV-uninfected controls [6]. Furthermore, among well-treated HIV-infected individuals ≥ 50 years without comorbidity or AIDS-defining events, the estimated median survival time remains lower than in the general population [7]. Importantly, cART is not able to eradicate HIV infection and the virus persists in long-lived reservoir cells [8] leading to the inevitable rebound of HIV within few days following cART interruption [9]. Thus, strengthened by recent scientific advances, the field is focused on development of strategies able to improve the immune control of HIV replication and to prolong the period of remission (i.e. HIV control without cART) leading to improvement of quality of life, decrease in morbidity and sparing costs.

Vaccines are essential elements of these functional cure strategies. The rationale of therapeutic immunization relies on the induction of HIV-specific cellular responses through the improvement of the magnitude of preexisting T-cell immune responses elicited by the natural infection which are not able to control HIV replication [10]. A common endpoint in vaccine trials is measurement of the breadth and magnitude of vaccine-induced CD4^+^ and CD8^+^ T-cell responses. Identification and characterization of CD8^+^ and CD4^+^ T-cell epitopes, as well as the restricting HLA alleles can play a major role in elucidating the nature of protective cellular responses and mechanism of the immune evasion of HIV. CD8^+^ T-cell epitopes are typically 8-11-mer peptides restricted by HLA class I [11] and CD4^+^ T-cell epitopes are 12-20-mer peptides restricted by HLA class II [12]. A large number of HIV epitopes have been identified and deposited into various databases. The growing amount of class-I and class-II HLA / peptide-binding data generated by experimental methods has supported the generation of more sophisticated, computational tools which can predict CD8^+^ and CD4^+^ T-cell epitopes within viral proteomes by using different data-driven bioinformatic approaches including Artificial Neural Network (ANN), Support Vector Machines, hidden Markov models, as well as other motif search algorithms. Using these HLA binding predictors can improve the efficiency of epitope mapping protocols in vaccine trials [13].

To achieve an optimal effect of therapeutic vaccination, the choice of antigens and vector is of critical importance. Several HIV-1 therapeutic candidate vaccines are currently under evaluation and one particularly considered approach is the use of dendritic cell (DC)-based vaccines [14]. Indeed, since DCs are the most powerful antigen-presenting cells (APCs) to initiate generation of antigen-specific CD4^+^ and CD8^+^ T-cell responses [15], it has been suggested that *ex vivo*-generated DCs might be the most potent activator of T-cell responses avoiding the use of potentially toxic adjuvants. The DALIA phase I trial evaluated a new DC-based therapeutic vaccine that consisted of *ex vivo*-generated IFNα DCs loaded with 5 HIV-1 lipopeptides in 19 cART-treated HIV-1 infected patients [16]. These lipopeptides (Gag 17–35, Gag 253–284, Nef 66–97, Nef 116–145 and Pol 325–355) were composed of HIV-1 clade B regions rich in CD8^+^ and CD4^+^ T-cell epitopes [17–18] that have shown induction of cellular immune responses in a phase II clinical trial in healthy volunteers [19]. We have shown that DALIA vaccination was well tolerated and increased the breadth and functionality of HIV-1 specific immune responses. Moreover, the DALIA study design included a 6-month analytic treatment interruption (ATI) which allowed to show an inverse correlation between the peak of viral load after ATI and the frequency of polyfunctional CD4^+^ T-cell responses after vaccination [20].

In the present study, we performed more in-depth epitope mapping to dissect anti-HIV-1 specific immune responses in order to better characterize vaccine responses associated with the control of viral load post-ATI. We showed that viral control was associated with the breadth and magnitude of IL-2 and IL-13 CD4^+^ T-cell responses targeting both immunodominant and subdominant HIV-1 peptides included in Gag, Pol and Nef vaccine regions. These results highlight the potential use of IL-13 as a new biomarker of HIV-1 vaccine efficacy and may help to better design the future DC therapeutic vaccine trials.

## Results

### DALIA vaccine regimen broadened breadth of HIV-specific immune responses

**Fig 1:**
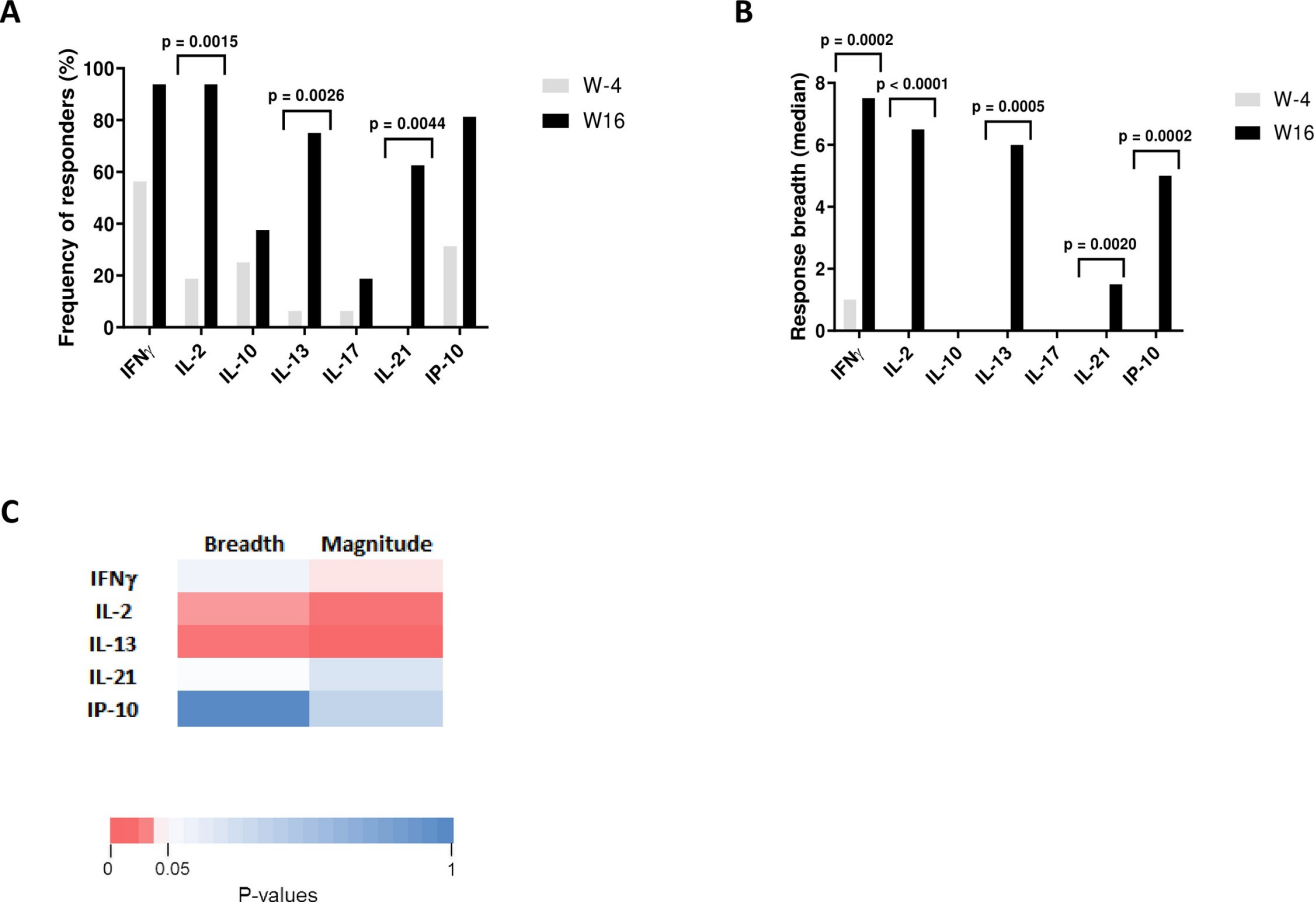
Immunogenicity of DALIA vaccine. PBMC from patients (n = 16) collected before (W-4) and after vaccination (W16) were stimulated with individual 15-mer peptide (n = 36) spanning the vaccine sequence. Cytokine production was measured in culture supernatant using a Luminex assay at 48 hours. **(A)** Comparison of frequency of responders for each cytokine at W-4 and W16 using McNemar’s test. A patient was considered responder when he had at least one positive response. Positivity threshold was defined for each cytokine as fluorescence intensity (FI) > 90th percentile of all Non-Stimulated (NS) values and > 3-fold background (NS condition). **(B)** Comparison of the breadth of the responses for each cytokine at W-4 and W16 (Median, Wilcoxon matched-pairs signed rank test). Response breadth was defined as the number of individual peptides inducing a positive response. **(C)** Heatmap showing Spearman P values for correlations between maximum of plasma HIV RNA measured in patients after ATI and cytokine responses quantified by Luminex assay after vaccination at W16 from supernatants of PBMC stimulated with individual 15-mer HIV peptides. Magnitude was defined as the sum of FI for all peptide-stimulations.

We previously showed that DALIA DC-based therapeutic vaccination (see Materials and Methods section and S1 Fig for study design) induced and increased HIV-1 specific CD4^+^ and CD8^+^ T-cell immune responses and a broad repertoire of cytokine producing cells [20]. To better characterize these responses, we analyzed the production of cytokines before (W-4) and after (W16) vaccination in PBMCs stimulated with individual 15-mer peptides (overlapping by 11 amino acids) spanning the LIPO-5 vaccine regions using Luminex technology. Positive responses were defined as production of at least one detectable cytokine following individual peptide stimulation (see Materials and Methods section and S1 Table). As shown in Fig 1A, at W16 a production of cytokines was observed in a higher proportion of patients as compared to baseline; IFNγ (94% at W16 / 56% at baseline), IL-2 (94% / 19%, p<0.05), IL-13 (75% / 6%, p<0.05), IL-21 (63% / 0%, p<0.05) and IP-10 (81% / 31%). As shown in Fig 1B, vaccination also significantly increased the breadth of responses. At baseline, the median number of epitopes inducing the production of at least one cytokine (IFNγ, IL-2, IL-10, IL-13, IL-17, IL-21 or IP-10) were between 0–1 (range 0–7). After vaccination, these numbers were significantly increased (p<0.05) for IFNγ, IL-2, IL-13, IL-21 and IP-10 (median between 1.5–7.5, range 0–28). In contrast, neither IL-10 nor IL-17 responses were significantly increased after vaccination (Fig 1A and 1B). Thus, DALIA vaccine regimen broadened breadth and improved functionality of HIV-specific immune responses targeting vaccine regions.

### Vaccine-elicited cytokine responses are associated with lower viral load following cART interruption

We next correlated the breadth and the magnitude (fluorescence intensity, FI) of PBMC responses to individual HIV 15-mer peptides after vaccination (W16) and peak of plasma HIV RNA in patients following cART interruption. We found an inverse correlation (Spearman) between the maximum of plasma HIV RNA values and the number of peptides eliciting IL-2 (r = -0.58, P = 0.020), and IL-13 (r = -0.66, P = 0.007) production, and the magnitude of the response for IFNγ (r = -0.51, P = 0.047), IL-2 (r = -0.66, P = 0.007) and IL-13 (r = -0.71, P = 0.003). No correlation was observed for IL-21 or IP-10 (Fig 1C). These results showed an association between the breadth and magnitude of vaccine-elicited functional responses at W16 and a lower viral rebound following ATI post vaccination.

### Identification of immunodominant HIV responses induced by the vaccine

**Fig 2:**
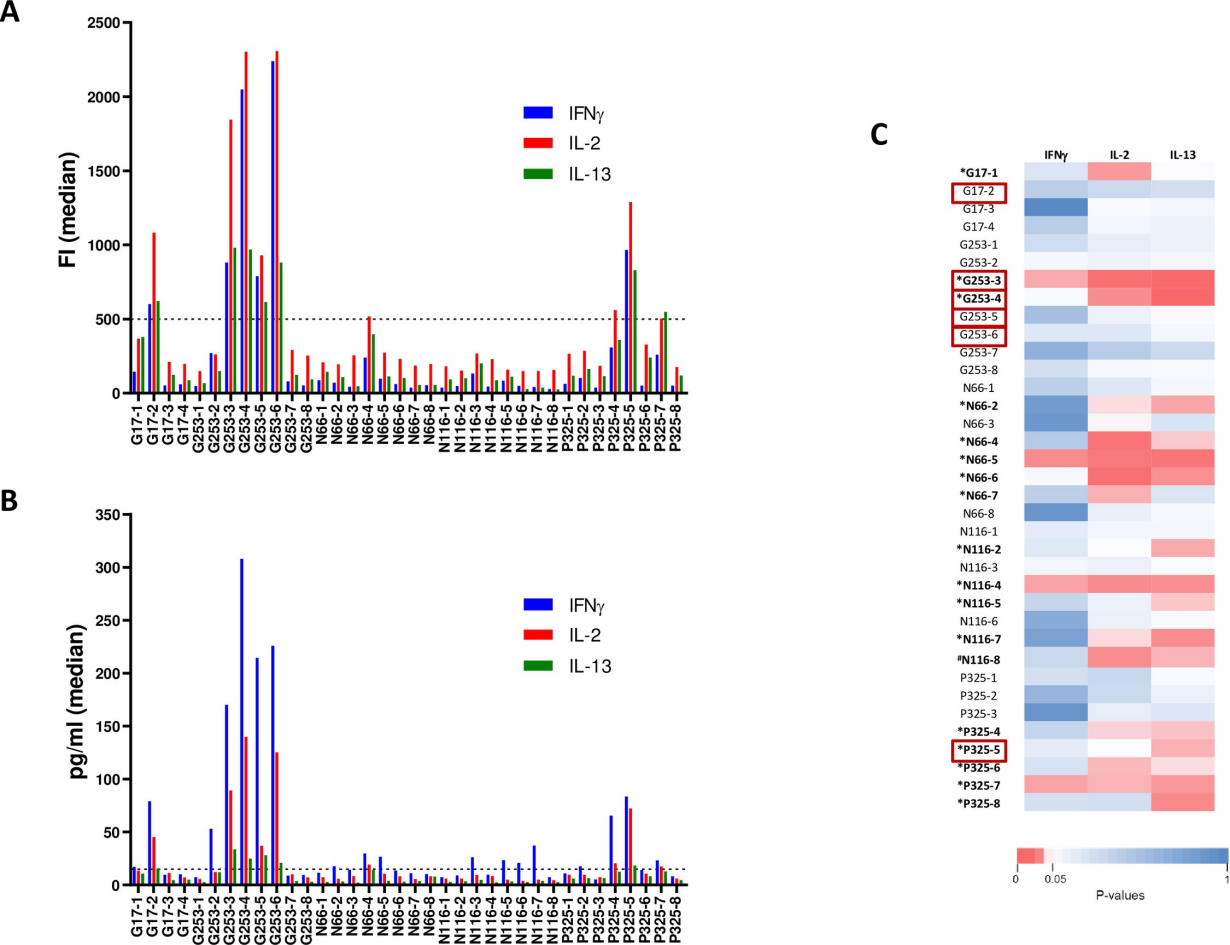
Magnitude of IFNγ, IL-2 and IL-13 responses at W16. PBMC from patients (n = 16) collected after vaccination (W16) were stimulated with individual 15-mer peptide (n = 36) spanning the vaccine sequence. Cytokine production was measured in culture supernatant using a Luminex assay at 48 hours. **(A)** Magnitude (FI) of IFNγ, IL-2 and IL-13 responses (median). Dotted line represents an arbitrary threshold of 500 FI for the definition of strong responses. **(B)** Magnitude (pg/ml) of IFNγ, IL-2 and IL-13 responses (median). Dotted line represents an arbitrary threshold of 15pg/ml for the definition of strong responses. **(C)** Heatmap showing Spearman P values for correlations between maximum of plasma HIV RNA measured in patients after ATI and IFNγ, IL-2 and IL-13 cytokine responses assessed by Luminex assay after stimulations of PBMC with individual 15-mer HIV peptides. *Peptides of interest showing at least one inverse correlation (in bold). ^#^Peptide showing at least one inverse correlation but did not passed positivity criteria described in methods section (in bold). Immunodominant peptides are highlighted within red rectangles.

Next, we sought to characterize peptide-specific IFNγ, IL-2 and IL-13 responses further, since they were significantly associated with lower viral load after ATI. Before vaccination (W-4), only a weak cytokine secretion was observed (S2 Fig). After vaccination (W16), we observed that each peptide induced a similar polyfunctional cytokine profile (Fig 2A) and that 6 peptides (Gag17-2, Gag253-3, Gag253-4, Gag253-5, Gag253-6, Pol325-5) induced a stronger response in at least half of the patients, either in FI (Fig 2A) or in concentration (Fig 2B). These immunodominant peptides were derived from the HIV-1 Gag and Pol vaccine sequences and not from Nef. Individual concentration data for all peptide stimulations are provided in S2 Table. No secretion of IFN-γ, IL-2 and IL-13 after vaccination was observed with non-stimulated PBMC or PBMC stimulated with non-vaccine-specific HIV-1 peptides (pools of 15-mer overlapping peptides from p2p6 Gag, outside the vaccine sequence) and high secretion of these cytokines was observed using SEB stimulation (S3 Fig).

### Identification of targeted peptides associated with lower viral load following cART interruption

We then investigated whether peptide-specific secretion of IFNγ, IL-2 and IL-13 cytokines observed after vaccination (W16) could be associated with viral dynamics after ATI. Thus, we correlated the magnitude of cytokine responses to the 36 individually tested peptides with the maximum viral load detected following cART interruption. For eighteen peptides, an inverse correlation (Spearman) between the maximum of HIV RNA values and magnitude of the specific response for at least one cytokine was found (Fig 2C, peptides in bold). All of them, except N116-8, passed positivity criteria described in Methods section (labeled with *). Thus, responses elicited by the vaccination against 17 peptides of interest from Gag, Nef and Pol proteins were identified as potentially associated with control of viral load post-ATI. Interestingly, there was an overlap with 3 (Gag253-3, Gag253-4, Pol325-5) of the 6 immunodominant peptides previously identified in Fig 2A and 2B (highlighted within red rectangles).

### Identification of HIV-specific cytokine-producing T cells

**Fig 3:**
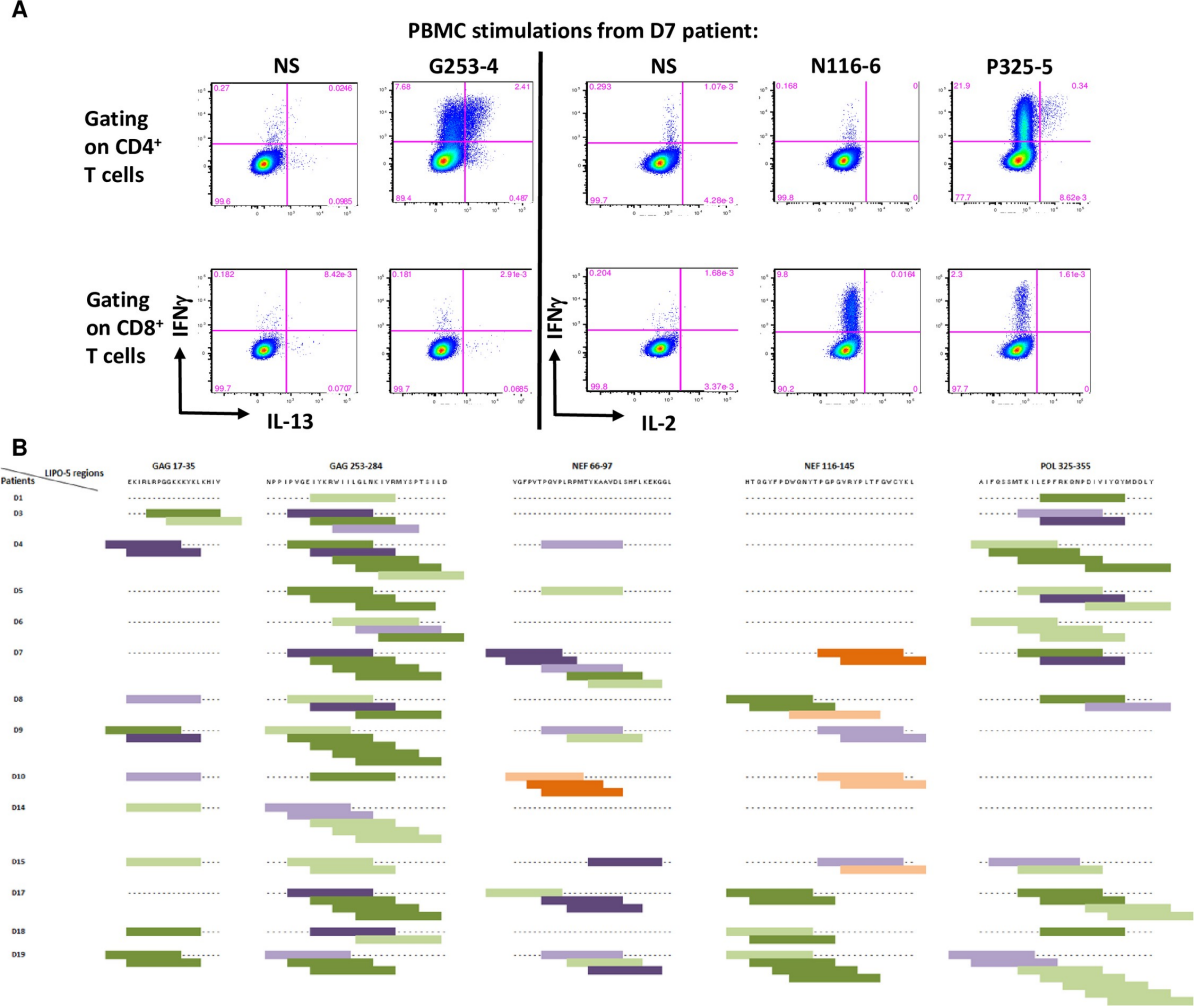
DALIA peptide vaccine coverage. PBMC from patients (n = 14) collected after vaccination (W16) were stimulated at day 0 with individual 15-mer peptides (previously inducing a positive response in Luminex assay), cultured with rIL-2, and restimulated by the same peptides at day 7. Cytokine production was measured using an ICS assay. **(A)** Cytometry dot plots of PBMC from one representative individual (D7 patient) stimulated with G253-4, N116-6 or P325-5 15-mer peptides. **(B)** Mapping of CD4^+^ and CD8^+^ T-cell responses observed for each of the 14 patients using 15-mer peptides. Light orange: low CD8^+^ T-cell responses, dark orange: high CD8^+^ T-cell responses, light green: low CD4^+^ T-cell responses, dark green: high CD4^+^ T-cell responses, light purple: low CD4^+^ and CD8^+^ T-cell responses, dark purple: high CD4^+^ and CD8^+^ T-cell responses. An ICS response was considered positive for a given cytokine if > 3-fold the negative control (unstimulated cells) and >0.05%. Cut off for high and low T-cell responses was defined < or > 5%.

Intracellular Cytokine Staining (ICS) were assessed to analyze post-vaccination HIV-specific CD4^+^ and CD8^+^ T-cell responses from 14 patients. Cytometry dot plots from one representative individual (D7 patient) showing an HIV-specific CD4^+^ T-cell response with G253-4 peptide stimulation (IFNγ and IL-13), an HIV-specific CD8^+^ T-cell response with N116-6 peptide stimulation (IFNγ and IL-2), and a dual HIV-specific CD4^+^ and CD8^+^ T-cell response with P325-5 peptide stimulation (IFNγ and IL-2) after vaccination are represented in Fig 3A. A total of 117 CD4^+^ and 46 CD8^+^ cytokine-producing T-cell responses directed to 29 and 22 peptides, respectively, were observed (Fig 3B). Although all vaccine regions induced CD4^+^ and CD8^+^ T-cell responses, Gag 253–284 was the main targeted region for CD4^+^ T-cell responses and Nef 66–97 was the main targeted region for CD8^+^ T-cell responses. Immunodominant HIV 15-mer peptides previously identified in Fig 2A and 2B induced both CD4^+^ and CD8^+^ T-cell responses, but CD4^+^ T-cell responses were more frequent. Moreover, targeted peptides associated with lower viral rebound after ATI induced either CD4^+^ T-cell responses alone (4/16 peptides) or both CD4^+^ and CD8^+^ T-cell responses (12/16 peptides).

Interestingly, despite the fact that some peptides were not predicted to bind HLA-DR molecules of the patients according to NetMHCIIpan 3.2 server [21], a significant frequency of CD4^+^ T-cell responses was observed (S4A Fig). For 61 of these non-predicted HLA-DR CD4^+^ T-cell responses, we used single HLA-DRB1 transfected cell lines as APC to try to identify the restricting DRB1 alleles involved in the IFNγ-positive CD4^+^ T-cell responses. We were able to identify a total of 8 new HLA-DR restricted CD4^+^ T-cell epitopes covering 14 (23%) of the 61 T-cell responses (Table 1 and S3 Table). These epitopes were localized in HIV-1 Gag, Nef and Pol vaccine sequences. Interestingly, 3 of them were immunodominant (Gag17-2, Gag253-3 and Pol325-5) and 5 of them were associated with a lower viral rebound after ATI (Gag253-3, Nef66-5, Nef66-6, Pol325-5, and Pol325-8).

**Table 1:** List of new HLA-DR-restricted CD4^+^ T-cell epitopes identified.

15-mer CD4^+^ T-cell epitope	Sequence	Restriction Class II HLA	Patient
Gag 17–2	EKIRLRPGGKKKYKL	DRB1*0301	D15 / D18
		DRB1*1301	D15
Gag 253–2	NPPIPVGEIYKRWII	DRB1*1301	D14
*Gag 253–3	PVGEIYKRWIILGLN	DRB1*0402	D5
		DRB1*1301	D14 / D15
Nef 66–1	GLTTMVGFPVTPQVP	DRB1*0401	D7
*Nef 66–5	RPMTYKAAVDLSHFL	DRB1*1401	D17
*Nef 66–6	YKAAVDLSHFLKEKG	DRB1*0401	D7
*Pol 325–5	EPFRKQNPDIVIYQY	DRB1*0101	D8 / D18
*Pol 325–8	YQYMDDLYVGSDLEI	DRB1*0405	D17

*Peptides associated with a lower viral rebound (in bold)

For CD8^+^ T cells, only few responses were observed in comparison to the high frequency of patients whose HLA class I molecules were predicted to bind to the different 15-mer peptides according to NetMHCpan 4.0 server [22] (S4B Fig). For 38 IFNγ-positive CD8^+^ T-cell responses observed and predicted, we used 8- to 10-mer peptides in ICS assays to identify the optimal CD8^+^ T-cell epitopes. A total of 30 CD8^+^ T-cell responses were identified (79%) of which 6 (20%) were directed towards an epitope shared by two different 15-mer peptides. So 24 unique IFNγ-positive CD8^+^ T-cell responses were observed, leading to the identification of a total of 18 different well-described CD8^+^ T-cell epitopes (Table 2).

**Table 2:** List of optimal CD8^+^ T-cell epitopes identified.

Optimal CD8^+^ T-cell epitope	Sequence	Restriction Class I HLA	Patient
Gag 18–27	KIRLRPGGKK	A*0301	D8
		A*0301	D10
Gag 20–28	RLRPGGKKK	A*3001	D4
Gag 21–29	LRPGGKKKY	C*0602	D9
Gag 259–267	GEIYKRWII	B*4001	D7
Gag 260–267	EIYKRWII	B*0801	D19
Nef 66–74	VGFPVTPQV	C*0304	D7
Nef 68–76	FPVTPQVPL	B*3501 / C*0304 / C*0401	D7
Nef 71–79	TPQVPLRPM	B*0702 / B*3501	D10
Nef 73–82	QVPLRPMTYK	A*0301	D10
		A*0301	D17
		A*1101	D19
Nef 83–91	AAVDLSHFL	C*0802 / C*1502	D17
Nef 84–92	AVDLSHFLK	A*1101	D15
		A*1101	D19
Nef 128–137	TPGPGVRYPL	B*0702	D8
Nef 134–141	RYPLTFGW	A*2301	D9
Nef 134–143	RYPLTFGWCY	B*1801	D15
Nef 135–143	YPLTFGWCY	B*3501	D7
		B*3501	D10
Pol 325–333	AIFQSSMTK	A*1101	D19
Pol 327–335	FQSSMTKIL	B*3906 / C*0701 / C*0702	D15
Pol 342–350	NPDIVIYQY	B*3501	D7
		B*3508	D5

Thus, DALIA vaccine regimen induced more CD4^+^ than CD8^+^ T-cell responses and 8 new HIV-1 HLA-DR-restricted CD4^+^ T-cell epitopes with their restricting HLA-DRB1 allele were identified.

### Characterization of HIV-specific cytokine-producing T cells

**Fig 4:**
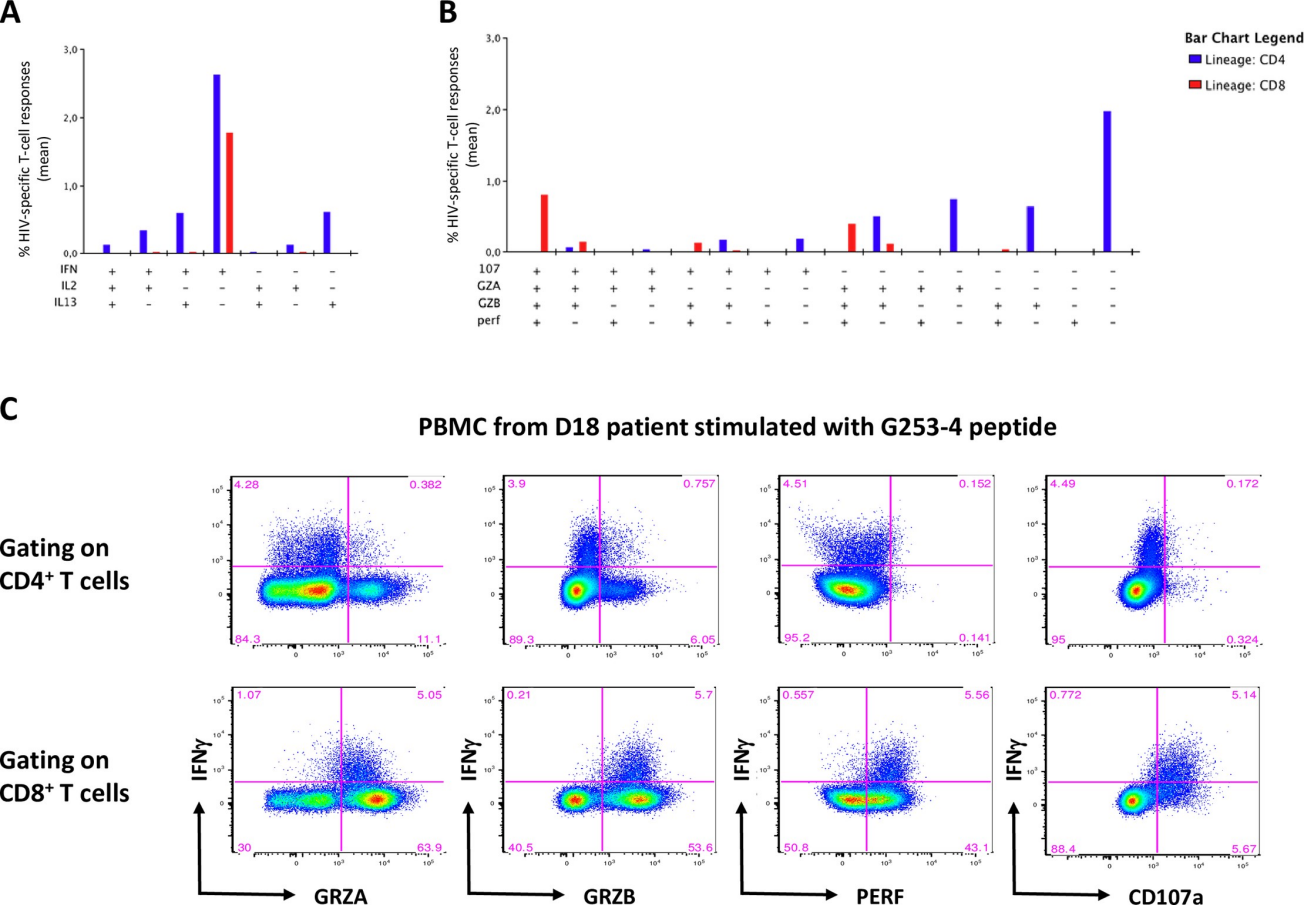
Identification and characterization of cytokine-producing cells by ICS assay. PBMC from patients (n = 14) collected after vaccination (W16) were stimulated at day 0 with individual 15-mer peptides (previously inducing a positive response in Luminex assay), cultured with rIL-2, and restimulated by the same peptides at day 7. Cytokine production was measured using an ICS assay. **(A)** Mean % of HIV-specific IFNγ and/or IL-2 and/or IL-13 CD3^+^CD56^-^CD4^+^ and CD3^+^CD56^-^CD8^+^ T cells to sixteen individual 15-mer HIV-1 peptides. **(B)** Mean % of cytotoxic marker-expressing cells among cytokine-positive HIV-1-specific CD3^+^CD56^-^CD4^+^ and CD3^+^CD56^-^CD8^+^ T cells to sixteen individual 15-mer HIV-1 peptides. **(C)** Cytometry dot plots from one representative individual (D18 patient) showing cytotoxic profile of HIV-1-specific CD4^+^ and CD8^+^ T cells after PBMC stimulation with G253-4 peptide.

Looking at the polyfunctionality of CD4^+^ and CD8^+^ T-cell responses induced by vaccination (W16) using ICS, we observed that vaccine-specific T cells were mainly monofunctional IFNγ^+^ (IFNγ^+^IL-2^-^IL-13^-^) CD4^+^ and CD8^+^ T cells, and to a lesser extent IL-13^+^ (IFNγ^-^IL-2^-^) CD4^+^ T cells. Some CD4^+^ T cells were able to produce 2 cytokines (IFNγ^+^IL-2^-^IL-13^+^ and IFNγ^+^IL-2^+^IL-13^-^ CD4^+^ T cells), and only few IFNγ^+^IL-2^+^IL-13^+^ and IFNγ^-^IL-2^+^IL-13^-^ CD4^+^ T cells were induced by vaccination (Fig 4A). Since cytotoxic CD4^+^ T cells have already been described in HIV-1 seropositive patients [23–24], we decided to characterize cytokine-positive CD4^+^ and CD8^+^ T cells using cytotoxic and degranulation markers such as granzyme A (GRZA), granzyme B (GRZB), perforin (Perf) and CD107a. We observed that CD4^+^ T-cells induced by vaccination were mainly non-cytotoxic (CD107a^-^GRZA^-^GRZB^-^Perf^-^), and to a lesser extent able to express GRZA and GRZB or CD107a, but not perforin. Conversely, CD8^+^ T cells exhibited mostly a cytotoxic phenotype (CD107a^+^GRZA^+^GRZB^+^Perf^+^ and CD107a^-^GRZA^+^GRZB^+^Perf^+^) (Fig 4B). Cytometry dot plots from one representative individual (D18 patient) showing cytotoxic profile of HIV-1-specific CD4^+^ and CD8^+^ T cells stimulated with G253-4 peptide after vaccination are represented in Fig 4C.

## Discussion

We report that vaccination with *ex vivo*-generated DCs pulsed with HIV-1 long peptides (HIV-1 lipopeptides) induced and/or expanded HIV-1-specific CD4^+^ T cells secreting IFNγ, IL-2 and IL-13, and also CD8^+^ T cells producing IFNγ, perforin and granzymes.

All five HIV-1 peptide regions included in the vaccine induced CD8^+^ T-cell responses. Previous data have shown that these HIV-derived long peptides were enriched in CD8^+^ T-cell epitopes and that lipopeptide vaccination either alone or combined with other agents induced CD8^+^ T-cell responses in HIV-negative or HIV-positive individuals [17,19,25]. Interestingly, we previously showed that the DALIA vaccine elicited predominantly IFNγ^+^ and TNFα^+^ CD8^+^ T cell responses [20], and here we showed that these CD8^+^ T cell responses also exhibit cytotoxicity markers, thus indicating a broader functional profile in contrast to those elicited by the administration of HIV-1 lipopeptides alone or in combination with a canarypox vector ALVAC [26]. Interestingly, a comparison of epitopes recognized by vaccine-elicited CD8^+^ T cells showed that among the 18 optimal CD8^+^ T-cell epitopes identified in our study, four belongs to the nine most promiscuously presented HIV epitopes described by Frahm and colleagues [27]. To provide global coverage of HIV-1 population diversity, mosaic vaccines containing conserved regions from different HIV-1 clades were developed [28]. It is noteworthy that RMYSPTSI, one of the five Gag conserved epitopes in the tHIVconsvX therapeutic vaccine, which had ability to suppress replication of circulating HIV-1 in HIV-1-infected individuals [29], was present in the DALIA vaccine sequence (Gag 253–284 region). Furthermore, three immunodominant HIV-1 clade B epitopes identified in our study have the ability to be recognized by CD8^+^ T cells from CRF02_AG-infected Ivorian patients [30] and two others were shown to be targeted by CTL from HIV-resistant Kenyan sex workers who had frequent exposure to HIV-1 clades A, C and D [31]. However, it is noteworthy that despite this broad repertoire and a highly cytotoxic phenotype of CD8^+^ T-cell responses, we did not observe any association between these responses and the control of HIV replication in patients following ATI. One limitation of our study is the lack of demonstration of the real killing capacity of these cells, which was precluded for technical reasons. Nevertheless, assuming that it is likely that a therapeutic epitope-based vaccine certainly needs to contain different CD8^+^ T-cell epitopes able to bind many different HLA haplotypes, in order to circumvent HIV-1 mutations, our data demonstrate that our DALIA platform is an efficient immunogenic vaccine strategy. Moreover, these data reinforce the need to combine immunotherapeutic strategies along with a vaccine to achieve a functional control of HIV replication.

Our in-depth analysis extended previous results characterizing the HIV-specific CD4^+^ T-cell responses elicited by the DALIA vaccine. These results are supported by several studies showing that robust HIV-1-specific CD4^+^ T-cell responses are associated with natural control of primary HIV-1 infection [32], predictive of persistent AIDS-free infection [33] and control of viremia in long-term non-progressors [34]. Furthermore, loss of non-progressor status was strongly associated with undetectable or declining Gag p24 CD4^+^ T-cell responses [35]. We described an inverse correlation between the breadth and magnitude of vaccine-induced IL-2 responses against individual 15-mer peptides and the maximum of viral load after ATI. The importance of IL-2 production by HIV-specific CD4^+^ T cells was already demonstrated, since IL-2 production by HIV-specific CD4^+^ T cells was shown to be associated with the persistence of a stable T-cell memory compartment [36], and HIV-specific CD4^+^ lymphoproliferative responses were shown to enhance *ex vivo* proliferative activity of HIV-1-specific CD8^+^ T cells [37]. Moreover, IFNγ^+^IL-2^+^ CD4^+^ T cells have been associated with control of viremia in HIV- seropositive patients [38–41], and Lu and colleagues found an inverse correlation between HIV-1 viral load and HIV-1-specific IFNγ and IL-2 producing CD4^+^ cells after vaccination of cART naïve HIV-1 individuals with a DC-based therapeutic vaccine pulsed with aldrithiol-2-inactivated HIV-1 [42]. Besides IL-2 responses, we also showed an inverse correlation between the breadth and magnitude of 15-mer peptides-mediated IL-13 responses and the maximum of viral load detected post-ATI. Similarly to the IL-2, we showed that IL-13 was mostly produced by non-cytotoxic CD4^+^ T cells. IL-13 is considered a Th2 cytokine and is poorly studied in the HIV field. However, it has recently been shown that HIV-specific Th2 responses could predict HIV vaccine efficacy [43] and that Th2 responses induced after SIV vaccination were correlated with a decrease risk of SIV acquisition [44]. We have already observed IL-13 secretion after vaccination of healthy volunteers with LIPO-5 [45] but to our knowledge, the only other publication studying IL-13 secretion in a therapeutic HIV vaccine context showed an association between higher IL-13 secretion after vaccination and higher viral load after ATI [46]. These discrepancies could be explained by a difference in vaccine composition (Gag/Pol/Nef lipopeptides-loaded activated DCs versus Gag p24 peptides + GM-CSF) and a difference in cytokine measurement protocol (48h stimulation with 15-mer peptides versus 6 days stimulation with recombinant Gag p24). In line with our results, it has been demonstrated that IL-13 inhibited HIV production in primary blood-derived human macrophages *in vitro* [47] and that IL-13 and IFNγ secretion by activated T cells in HIV-1 infection was associated with viral suppression and a lack of disease progression [48]. Moreover, IL-13 was shown to increase HIV-specific and recall responses from HIV-1-infected subjects *in vitro* by modulating monocytes [49]. Last but not least, IL-13 was also shown to be produced by seronegative subjects highly exposed to HIV [50].

All five HIV-1 lipopeptide sequences used to load the DALIA vaccine, in particular Gag 253–284 and Pol 325–355 regions, induced strong CD4^+^ T-cell responses. Accordingly, these five HIV regions were shown to induce CD4^+^ T-cell responses in non-HIV (19) or HIV-infected patients [25] vaccinated with HIV-1 lipopeptides, and some epitopes contained in these vaccine regions were previously described as present in a set of 11 immunogenic HLA-DR supertype CD4^+^ epitopes [51] or 31 immunodominant CD4^+^ T-cell epitopes [52]. We extended this repertoire by identification of 6 immunodominant 15-mer peptides elicited after DALIA vaccination: Gag17-2 (EKIRLRPGGKKKYKL), Gag253-3 (PVGEIYKRWIILGLN), Gag253-4 (IYKRWIILGLNKIVR), Gag253-5 (WIILGLKNIVRMYSP), Gag253-6 (GLNKIVRMYSPTSIL) and Pol325-5 (EPFRKQNPDIVIYQY). The first five were already described as CD4^+^ T-cell epitopes [53] and listed in the HIV molecular immunology database (http://www.hiv.lanl.gov/content/immunology/tables/helper_summary.html). Gag253-3, Gag253-4, Gag253-5 and Gag253-6 have been described as DRB1*04:01 restricted epitopes in a study using HLA DR4 transgenic mice [54]. Gag253-4, Gag253-5 and Gag253-6 were also found to be promiscuous peptides, i.e. able to bind multiple HLA-DR alleles, using NetMHCIIpan 3.2, the server we used to predict binding of 15-mer peptides to HLA Class II DRB1 alleles. Pol325-5 was not listed in the HIV molecular immunology database, nonetheless it is nearly totally contained (93% overlap) in a Pol CD4^+^ T-cell promiscuous epitope identified by van der Burg and colleagues [55]. Of note, in some studies where the breadth of Gag-specific CD4^+^ T-cell responses was shown to be associated with lower viral load in HIV-infected individuals [56–58], Gag17-4, Gag253-4, Gag253-5, Gag253-6 and Gag253-7 were the main targeted epitopes. HIV-1 Nef-specific CD4^+^ T-cell responses are also important to control HIV-1 viral load and the Nef 66–97 peptide included in the DALIA vaccine was found among the 4 Nef epitopes previously associated with non-progression in HIV-1 infection [59]. In our study, we found that responses to seventeen 15-mer peptides of interest were potentially associated with lower viral load after ATI. Among them, Gag253-4 and Nef66-6 CD4^+^ T-cell epitopes were found in the studies mentioned above. All these data suggest that Gag253-4 is probably the most important HIV-1 sequence to be included in a therapeutic vaccine, and this is strengthened by its large capacity to bind to the 20 most frequent HLA-DRB1 alleles in the world population [60].

To study the reliability of the epitope prediction servers used in our study, we first looked whether the observed responses were predicted. Among the 117 cytokine-positive CD4^+^ T-cell responses detected in our study, only 35% were predicted to bind DRB1 molecules by NetMHCIIpan 3.2 server, but it is difficult to comment on the reliability of NetMHCIIpan 3.2 server because we only used HLA-DRB1 (and not HLA-DP or -DQ) molecules of the patients for epitope prediction with this server. By contrast, all 46 observed CD8^+^ T-cell responses were predicted to bind HLA class I A, B or C molecules of the patients by NetMHCpan 4.0 server. Then we looked whether the predicted responses were actually observed. Interestingly, only 10% of all predicted CD8^+^ T-cell responses were observed. A potential explanation could be that even the servers used in this study predict binding affinity of peptides to MHC molecules, affinity is only one of the determinant factors for a peptide to induce an immune response. Indeed, to be immunogenic, a peptide needs to be efficiently processed by the cell machinery and then presented by MHC molecules. Epitope flanking residues can influence the proteasome or peptidase processing of the antigen. TCR affinity for the peptide/MHC complex also has an important role on the induction of immune responses. For CD4^+^ T-cell responses, 51% of predicted responses were observed and we found 3 different patterns: 1) some peptides were predicted to bind HLA-DR molecules frequently expressed by the patients and for those we observed the majority of CD4^+^ T-cell responses (this was the case for Gag253-4/5/6); 2) some peptides were predicted to bind HLA-DR frequently expressed by the patients and for those we observed only few CD4^+^ T-cell responses (this was the case for Gag17-4, Gag253-7 and Pol325-1/2); 3) some peptides were not predicted to bind HLA-DR molecules expressed by the patients, but for those we nevertheless observed a CD4^+^ T-cell responses (this was the case for Gag253-3, Nef66-4 and Pol325-4/5). This latter result could easily be explained by the fact that these peptides could bind HLA-DP or -DQ alleles. However, another explanation could be that HIV-1 regions that bind some HLA class II alleles very weakly can induce CD4^+^ T-cell responses, and this is actually the case for the HIV-1 lipopeptides used in the DALIA vaccine, for which the hierarchy of CD4^+^ T-cell epitopes relies on the frequency of pre-existing peptide-specific T cells in healthy donors [61].

Recently, an association of HLA-DRB1 allele expression with HIV viral load profiles at a population level was reported [62]. DRB1*15:02 was shown to be associated with HIV control whereas DRB1*03:01 was shown to be associated with HIV progression. Here, we identified using a modified ICS protocol with single HLA DRB1 transfected cell lines as APC, in vaccinated patients, 8 new HLA-DR CD4^+^ T-cell epitopes restricted by different HLA-DRB1 alleles. Among them, Gag17-2 was restricted by DRB1*03:01. Although, in our study, due to the small number of patients, we could not find any effect of these two HLA-DRB1 alleles on viral load dynamics after ATI, prediction analysis revealed that only two epitopes Gag253-4 and Gag253-5 could bind the DRB1*03:01 allele while Gag253-4, Gag253-5, Gag253-6, Gag253-7, Pol325-1, Pol325-2 and Pol325-7 could bind the “protective” DRB1*15:02 allele.

Our study has characterized epitope responses to a therapeutic HIV-1 vaccine beyond the “classical” analyses of responses to global HIV peptide pools. Our analysis benefited from the well described definition of HIV-1 peptides used for loading of the DC vaccine. This originality represents an advantage compared to similar studies using chemically or temperature inactivated whole autologous HIV or RNA-based pulsed DCs [42,63,64]. Regarding the need to significantly improve the breadth and the functionality of vaccine responses with the aim to better control HIV in the perspective of functional HIV cure, it is key to better identify the epitopes of interest. In contrast to several studies of these responses in natural infection, fine characterization of vaccine response at the level of individual peptides contained in therapeutic vaccine is lacking. We confirmed at the epitope level that the DALIA vaccine regimen induced CD8^+^ and CD4^+^ T-cell responses, and that IL-2 and IL-13 responses induced by the vaccine were mediated by CD4^+^ T cells. Beyond the dissection of precise epitopes, we described an inverse correlation between the breadth and magnitude of peculiar CD4^+^ T-cell responses producing IL-2 and/or IL-13 and a partial control of viral load after ATI.

From a clinical stand point, these results may help to better design the future planned DC therapeutic vaccine trial. First, it is likely that a combined strategy to complement CD8^+^ T-cell responses (immune checkpoint blockers, cytokines such as IL-7 or IL-15) to CD4^+^ elicited T-cell responses is warranted. Second, a particular attention will be paid to IL-2 and IL-13 CD4^+^ T-cell responses in relation to viral load dynamics after ATI. Since DC-based vaccines are difficult to make and will be challenging to use on a large scale, a new generation of vaccines targeting DC *in vivo* was developed using monoclonal antibodies directed against cell-surface receptors [65]. Thus, the sequences of the 5 lipopeptides used in the DALIA vaccine were recently fused with an anti-human CD40 antibody to target DCs *in vivo*. Targeting DC of HIV-1 infected patients *in vitro* with this new candidate vaccine (αCD40.HIV5pep) showed induction of CD4^+^ T-cell responses towards Gag 253–284 and Pol 325–355 regions in a majority of patients [66]. Safety and immunogenicity of this vaccine construct administrated intradermally with poly-ICLC was recently demonstrated in rhesus macaques [67]. Moreover, in humanized mice infected with HIV-1 and treated with antiviral drugs, αCD40.HIV5pep plus poly-ICLC vaccination rescued HIV-1-specific CD4^+^ and CD8^+^ T-cell responses and reduced the size of the HIV-1 reservoir, leading to significant control of HIV-1 rebound after cART interruption [68]. Thus, either *ex vivo* (DALIA) and *in vivo* (anti-CD40.HIV5pep) DC-targeting platforms are promising immune interventions to combine with cytokines, immune checkpoint inhibitors, as well as broad neutralizing antibodies for an HIV cure [69].

## Materials and methods

### Study design and vaccine preparation

The ANRS/VRI DALIA was a phase I single-center single-arm clinical trial (North Texas Infectious Diseases Consultants, Dallas, TX) sponsored by Baylor Institute for Immunology Research and the French Agency INSERM-Agence Nationale de Recherches sur le SIDA et les hepatites (INSERM-ANRS). Eligible participants were asymptomatic HIV-1-infected adults with CD4+ T cell counts >500 cells/μL and ≥25% of total lymphocytes, CD4 nadir ≥300 cells/μL, and no history of AIDS-defining events. Participants had to be on combination antiretroviral therapy (cART) with plasma HIV RNA <50 copies/mL at screening and within the previous 3 months. Participants received four vaccinations at weeks (W) 0, 4, 8 and 12 while on cART. Participants who had HIV-1 RNA <400 copies/mL at W22 were proposed to interrupt cART at W24 until W48. Flowchart describing the ANRS/VRI DALIA clinical trial, as well as baseline characteristics of the patients, have been reported previously [20] and design of the DALIA trial is shown in S1 Fig. Twenty participants were screened and 19 enrolled in the trial between 2009 and 2010. Among these 19 participants, three were not included in the present analyses due to lack of cryopreserved PBMCs to perform T-cell assays.

Blood monocytes from each patient were obtained from an apheresis at W-4 while on cART. DC-based vaccines were generated by culturing blood monocytes with GM-CSF and IFNα for three days. Differentiating DCs were then pulsed for 24 hours with lipopeptides (ANRS HIV-LIPO-5): HIV-1 LAI (clade B) Gag 17–35, Gag 253–284, Pol 325–355, Nef 66–97 and Nef 116–145, activated during the last 6 hours with lipopolysaccharide (LPS), purified under Good Manufacturing Practice guidelines, harvested and then frozen. Preclinical validation of the preparation and functional characterization of this vaccine has been previously reported [16]. Approximately 15x10^6^ viable frozen-thawed HIV lipopeptide-loaded DC were injected subcutaneously in 3 separate injection sites (3.3 ml per site) in the upper and lower extremities at each vaccination time point.

### Ethics statement

The trial was approved by the IRB of Baylor Research Institute (BRI) and registered on ClinicalTrials.gov (NCT00796770). All participants provided written informed consent.

### HLA typing

Samples were sent to LABS Inc (Centennial CO, USA) for HLA Typing (A, B, C, DRB1 and DQB1) using High Resolution Sequence Based Testing (SBT). HLA characteristics of participants (A, B, and C for HLA class I and DRB1 for HLA class II) are reported in S4 Table.

### Peptides used in T-cell assays

Fifteen-mer peptides (n = 36) overlapping by 11 amino acids (aa) and covering the vaccine sequences were synthesized by Biosynthesis (Lewisville, TX, USA) and used at a final concentration of 2 μM (sequences are listed in S1 Table). Eight- to ten-mer peptides synthesized by NeoMPS (now PolyPeptide Laboratories France, Strasbourg) were used at 2 μg/ml to identify optimal CD8^+^ T-cell epitopes.

### Prediction of CD4^+^ and CD8^+^ T-cell epitopes

NetMHCIIpan 3.2 [21] server was used to predict binding of peptides to MHC class-II DRB1 molecules of the participants and their putative CD4^+^ T-cell responses. NetMHCpan 4.0 [22] was used to predict binding of peptides to MHC class-I (A, B and C) molecules of the participants and potential CD8^+^ T-cell epitopes. These prediction methods are based on ANN. For NetMHCIIpan 3.2, the prediction values are given in IC50 values (in nM) and as %Rank. The percentile rank for a peptide is generated by comparing its score against the scores of 200,000 random natural peptides of the same length of the query peptide. Strong and weak binding peptides are identified based on %Rank, with customizable thresholds. We applied the default thresholds of 2% and 10% for strong and weak binders, respectively. For NetMHCpan 4.0, the method is trained on a combination of more than 180,000 quantitative binding data and mass spectrometry derived MHC eluted ligands. Rank of the predicted affinity was compared to a set of random natural peptides. The peptide will be identified as a strong binder if the % Rank is below the specified threshold for the strong binders, by default 0.5%. The peptide will be identified as a weak binder if the % Rank is above the threshold of the strong binders but below the specified threshold for the weak binders, by default 2%.

### Luminex assay

Frozen PBMCs from aphereses before (W-4) and after (W16) vaccination were thawed and rested at 37°C for 1h. Samples were filtered and resuspended in RPMI 1640 media supplemented with 10% Human serum (HS). Plates containing individual overlapping peptides, positive (Staphylococcal enterotoxin B (SEB)) and negative (no peptide) controls, were thawed at room temperature and 1x10^6^ cells were added in each well to a final volume of 300 μl. After 48h of culture, the plates were centrifuged, and 200 μl of each supernatant was collected and frozen at -80°C for cytokine detection. Measurements and analyses of secreted IL-2, IL-10, IL-13, IL-17, IL-21, IFNγ and IP10 (expressed in fluorescence intensity (FI) and concentration) were performed using an “in house” multiplex bead-based technology (Luminex) assay with a Bio-Plex 200 instrument (Bio-Rad) after a two-fold dilution of supernatants. Human IFNγ, IL-2, IL-10, IL-13, IL-17, IL-21 and IP-10 monoclonal antibody reagent pairs were developed and validated by the Baylor Institute for Immunology Research (BIIR). These pairs were conjugated to Luminex X-MAP SeroMAP microspheres according to manufacturer’s guidelines (Luminex). Biotinylation was performed with EZ link Sulfo-NHS LC Biotin from Thermo Fisher. PhycoLink Streptavidin-RPE was purchased from Prozyme. This multiplex was assessed using a commercial 68-Plex cytokine standard cocktail produced expressly for the BIIR by BioLegend with protocols comparable to that for Millipore multiplex processing. The BIIR Luminex Core facility maintains compliance with the External Quality Assurance Program Oversight Laboratory, a National Institutes of Health and National Institute of Allergy and Infectious Diseases Division of AIDS program for quality assessment review and ratings of laboratories involved in HIV/AIDS research and vaccine trials around the world.

### Intracellular Cytokine Staining (ICS)

Frozen PBMC from the apheresis at W16 were thawed and rested at 37°C for 3h in RPMI 1640 media with L-Glutamax supplemented with Penicillin / Streptomycin and 10% HS (R-10HS). On day 0 (D0), 2x10^6^ cells were cultured in 24 flat bottom wells culture plate with or without 2 μM of 15-mer peptides that induced a positive response by Luminex assay. IL-2 (10 U/ml) was added on day 2 and half of the volume of each culture well was refreshed with fresh media containing IL-2 (10 U/ml) at day 4. On day 7 the cells were divided in two 5 ml polypropylene tubes and washed two times. D0 non-stimulated cells were then stimulated or not with SEB, and D0 stimulated cells were re-stimulated or not with the same peptide as D0, in a 500 μl R-10HS final volume. For the cytotoxic panel, CD107a was also added during cell stimulation. One hour later, BD GolgiPlug and GolgiStop (Becton Dickinson France) were added and the culture was continued for additional 5 hours. Cells were then washed and stained for surface antibodies and live/dead marker for 20 minutes. Following fixation and permeabilization, cells were stained with intracellular antibodies for 30 minutes. Cells were then washed and resuspended in 250 μl PBS 1% PFA.

To determine optimal CD8 responses, predicted CD8^+^ T-cell epitopes (8- to 10-mers) were used in ICS assay.

To determine the DRB1 HLA molecules involved in the observed CD4^+^ T-cell responses, 14 different DRB1 (DRB1*01:01, 03:01, 04:01, 04:02, 04:03, 04:04, 04:05, 07:01, 11:01, 11:04, 13:01, 13:02, 14:01, 15:01) single transfected DAP.3 cell lines (LJI, USA) were pulsed for 1h with 15-mer peptides, washed four times and then added at D7 to D0-stimulated cells. Non-transfected DAP.3 cell line was used as negative control.

Two different ICS panels were tested, a first one for cytokine responses using anti-CD3 A700 (BD ref 557943), -CD4 BV605 (BD ref 562658), -CD8 efluor780 (eBioscience ref 9047-0087-120), -CD56 PECF594 (BD ref 564849), -IL-13 PE (BD ref 340508), -IFNγ PerCPCy5.5 (BD ref 560704), -IL-2 FITC (BD ref 559361), -IL-21 AF647 (BD ref 560493), Live dead fixable Aqua Dead (Life Technologies ref L34957), and a second for cytotoxic characterization of cytokine responses using anti-CD3 BV605 (Biolegend ref 300460), -CD4 BV785 (Biolegend ref 300554), -CD8 APC-H7 (BD ref 560179), -CD56 PC7 (BD ref 557747), -IL-13 PE (BD ref 340508), -IFNγ PECF594 (BD ref 562392), -IL-2 APC (eBioscience ref 17-7029-41), -Granzyme A Pacific Blue (Biolegend ref 507207), -Granzyme B AF700 (BD ref 560213), -Perforin PerCPCy5.5 (Biolegend ref 353314), -CD107a FITC (BD ref 555800), Live dead fixable Aqua Dead (Life Technologies ref L34957).

Samples were analyzed with a BD LSRII and data were acquired with DIVA software. FlowJo 9.9.6 (TreeStar, Inc., Ashland, OR), Pestle 1.7 and Spice 5.35 (M. Roederer, National Institute of Health) softwares were used for data analysis.

### Statistical analyses

For a given cytokine, a Luminex quantification was considered as a positive response if the fluorescence intensity (FI) was > 90^th^ percentile of all negative controls (non-stimulated cells before and after vaccination for all participants) and > 3 times the background (median of three negative control wells for the considered participant and time point). An ICS response was considered positive for a given cytokine if the frequency of stimulated CD3^+^CD56^-^CD4^+^ or CD3^+^CD56^-^CD8^+^ cells was > 3 times the negative control (unstimulated cells) and >0.05%.

Correlations between immunogenicity outcome at W16 and the maximum of HIV-1 RNA observed during ATI were analyzed by Spearman rank correlations. Comparison between frequency of responders before and after vaccination was performed using McNemar’s test and comparison between the breadth of the response observed before and after vaccination was performed using Wilcoxon matched-pairs signed rank test.

Statistical analyses were carried out using GraphPad Prism (version 8.0.1, GraphPad Software, San Diego, USA).

## Supporting information

S1 Fig Design of the DALIA trial. HIV-infected patients have been vaccinated at 0, 4, 8, 12 weeks and antiretroviral treatments have been interrupted at 24 weeks. Apheresis has been performed at 2 to 4 weeks (−2/−4) before the first vaccine injection (week 0), and 16 weeks and 48 weeks thereafter. Blood draw has been performed at each single visit. Of 20 eligible HIV-infected patients, 19 completed the vaccination and 16 reached the 48 weeks visit without resuming their antiretroviral treatment after interrupting it at 24 weeks.
(TIF)

S2 Fig Magnitude of IFNγ, IL-2 and IL-13 responses at W-4. Magnitude (FI) of IFNγ, IL-2 and IL-13 responses (median) quantified by Luminex assay after a 48h stimulation of PBMC with individual peptides. Dotted line represents the strong responses threshold defined in Fig 2A.
(TIF)

S3 Fig Magnitude of IFNγ, IL-2 and IL-13 responses for controls at W16. Magnitude (FI) of IFNγ, IL-2 and IL-13 responses (median) quantified by Luminex assay after a 48h stimulation of PBMC with medium alone (3 wells of non-stimulated cells), 3 non-LIPO-5 peptide pools from Gag p2p6 protein, or SEB. Dotted line represents the strong responses threshold defined in Fig 2A.
(TIF)

S4 Fig Predicted versus Observed T-cell responses. **(A)** CD4^+^ T-cell responses according to NetMHCIIpan 3.2 HLA-DRB1-binding predicted 15-mer peptides (blue line) or observed after 7-day ICS (green bars) for the 14 patients tested at W16. **(B)** CD8^+^ T-cell responses according to NetMHCpan 4.0 HLA-A/B/C-binding predicted 15-mer peptides (blue line) or observed after 7-day ICS (orange bars) for the 14 patients tested at W16.
(TIF)

S1 Table Peptide sequences. (TIF)

S2 Table Individual data of IFNγ, IL-2 and IL-13 concentration level (pg/ml) at W16. Luminex assay was performed after a 48h stimulation of PBMC with 36 individual 15-mer peptides. Absence of data means < LLOQ (lower limit of quantification). Positive responses identified using our positivity cut off based on FI are highlighted in yellow.
(XLSX)

S3 Table Identification of the HLA-DR molecules involved in the CD4^+^ T-cell responses using HLA-DRB1-transfected cell lines. PBMC were stimulated at day 0 with individual 15-mer peptides and cultured during 7 days with rIL-2. ICS assay was performed at day 7 after a 6h restimulation with 15-mer peptides or with HLA-DRB1-transfected cell lines previously pulsed (P) 1 hour with the 15-mer peptides. Non-restimulated PBMC, untransfected DAP.3 cell line pulsed 1 hour with the 15-mer peptides, and HLA-DRB1-transfected cell lines not pulsed (NP) with the 15-mer peptides were used as negative controls. An ICS response was considered positive (highlighted in bold in the table) if the frequency of stimulated CD3^+^CD56^-^CD4^+^ cells were > 3-fold the unstimulated cells and > 0.05%. Positive responses not predicted by NetMHCIIpan 3.2 are highlighted in yellow.
(XLSX)

S4 Table HLA characteristics of participants. (TIF)
