# Supplementary figures and images for: Anti-HIV potency of T-cell responses elicited by dendritic cell therapeutic vaccination
Source: PLoS Pathog. 2019 Sep 9;15(9):e1008011. doi: 10.1371/journal.ppat.1008011 (PMC6733439; doi:10.1371/journal.ppat.1008011)

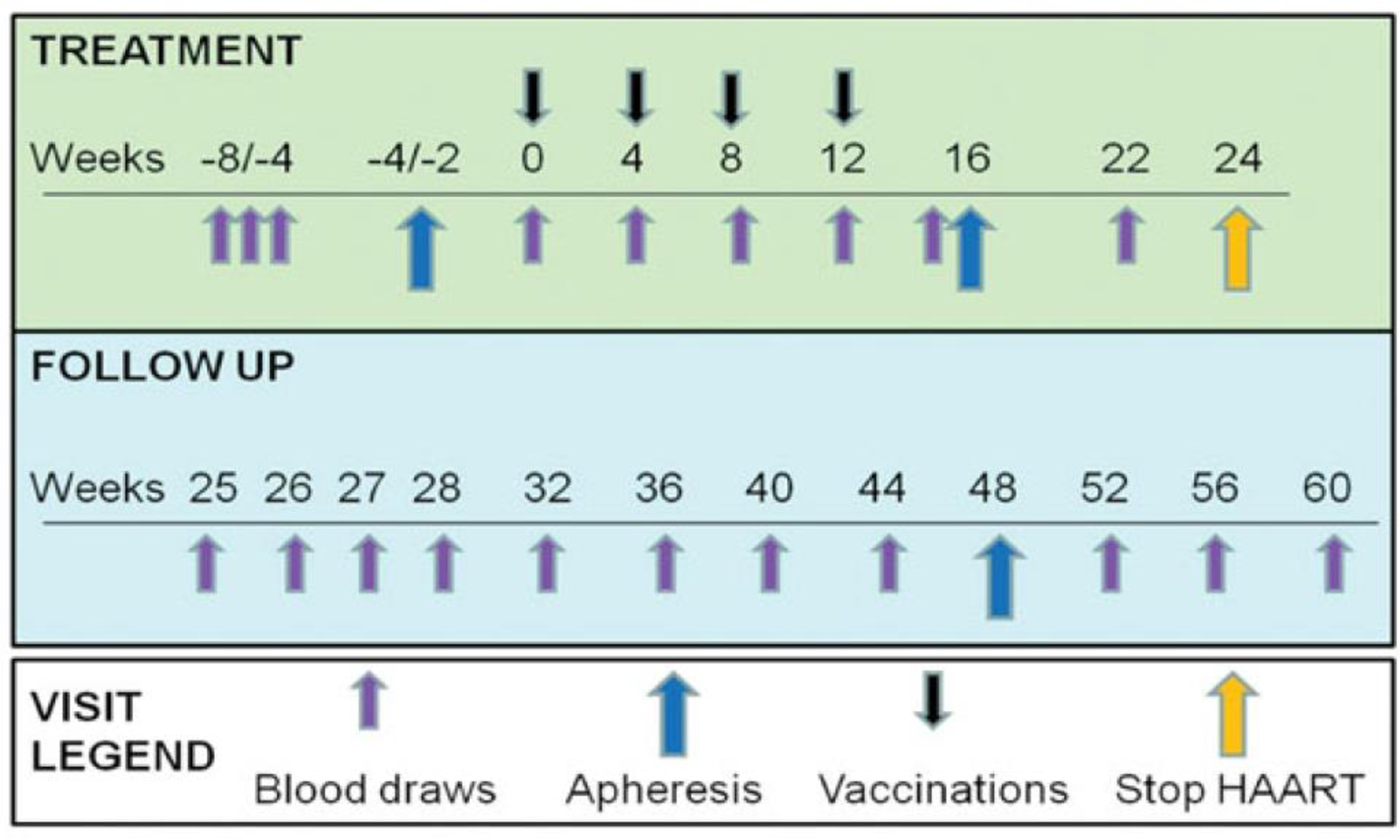

Supplement: S1 Fig — HIV-infected patients have been vaccinated at 0, 4, 8, 12 weeks and antiretroviral treatments have been interrupted at 24 weeks. Apheresis has been performed at 2 to 4 weeks (−2/−4) before the first vaccine injection (week 0), and 16 weeks and 48 weeks thereafter. Blood draw has been performed at each single visit. Of 20 eligible HIV-infected patients, 19 completed the vaccination and 16 reached the 48 weeks visit without resuming their antiretroviral treatment after interrupting it at 24 weeks. (TIF) [file ppat.1008011.s001.tif]

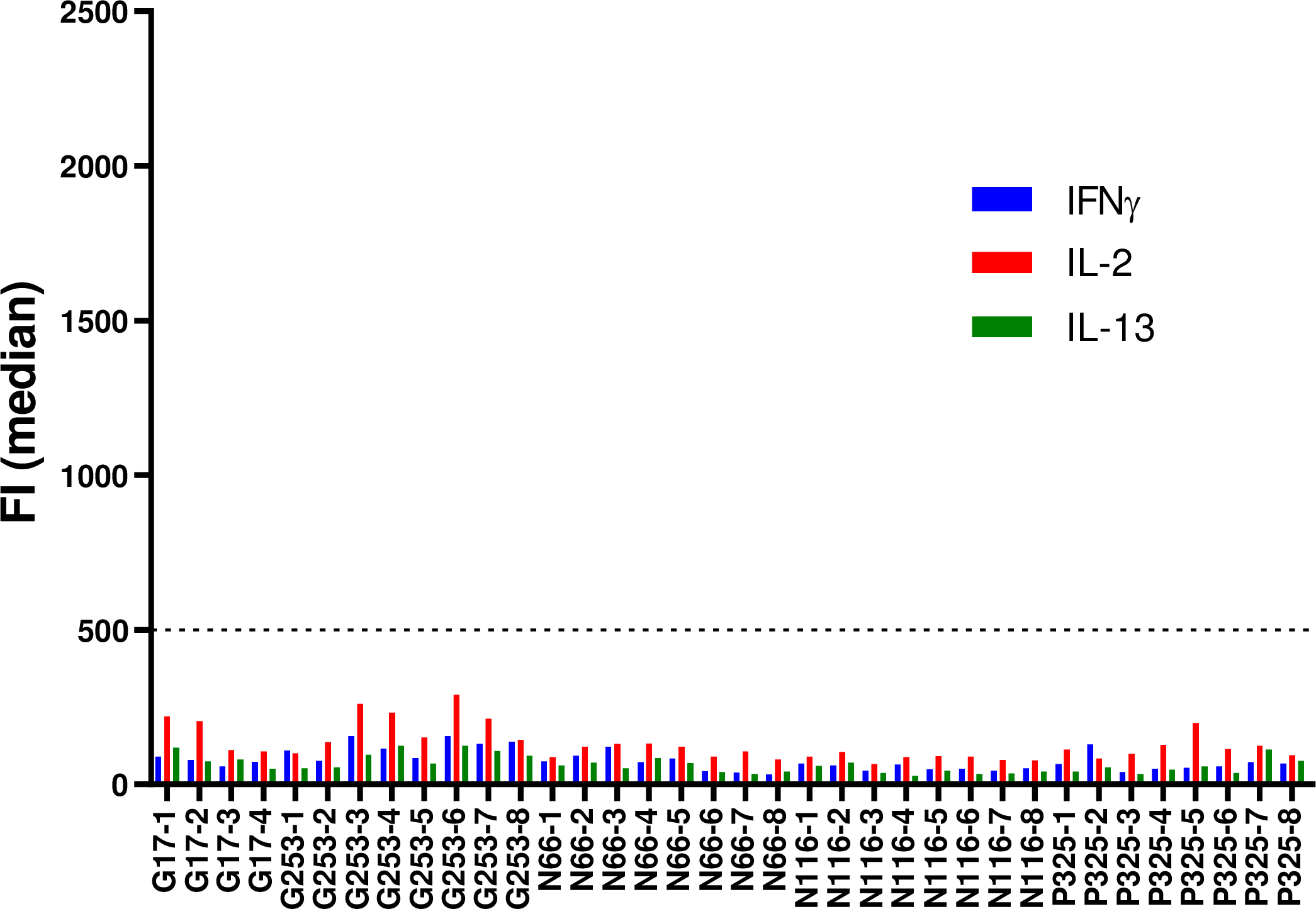

Supplement: S2 Fig — Magnitude (FI) of IFNγ, IL-2 and IL-13 responses (median) quantified by Luminex assay after a 48h stimulation of PBMC with individual peptides. Dotted line represents the strong responses threshold defined in Fig 2A. (TIF) [file ppat.1008011.s002.tif]

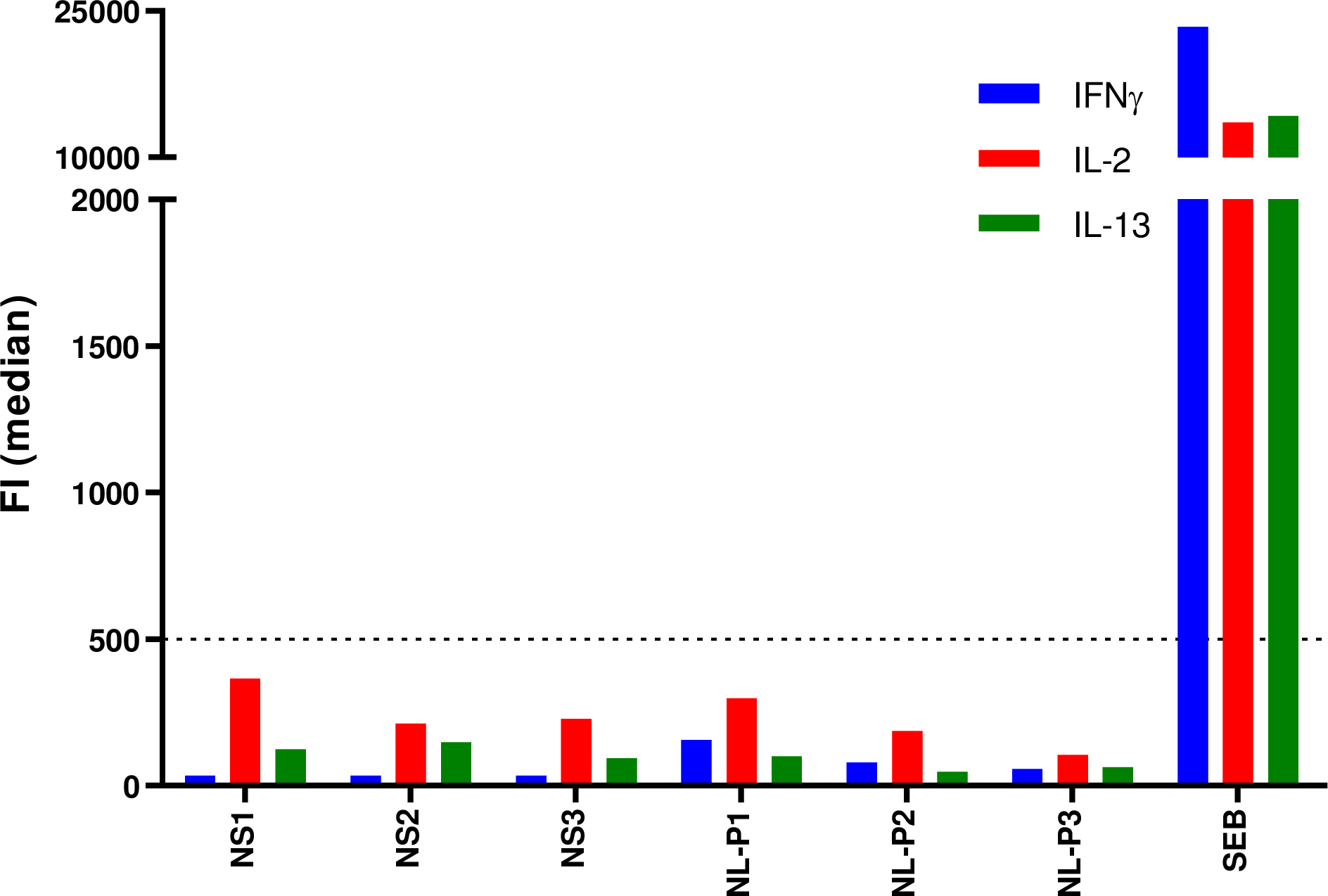

Supplement: S3 Fig — Magnitude (FI) of IFNγ, IL-2 and IL-13 responses (median) quantified by Luminex assay after a 48h stimulation of PBMC with medium alone (3 wells of non-stimulated cells), 3 non-LIPO-5 peptide pools from Gag p2p6 protein, or SEB. Dotted line represents the strong responses threshold defined in Fig 2A. (TIF) [file ppat.1008011.s003.tif]

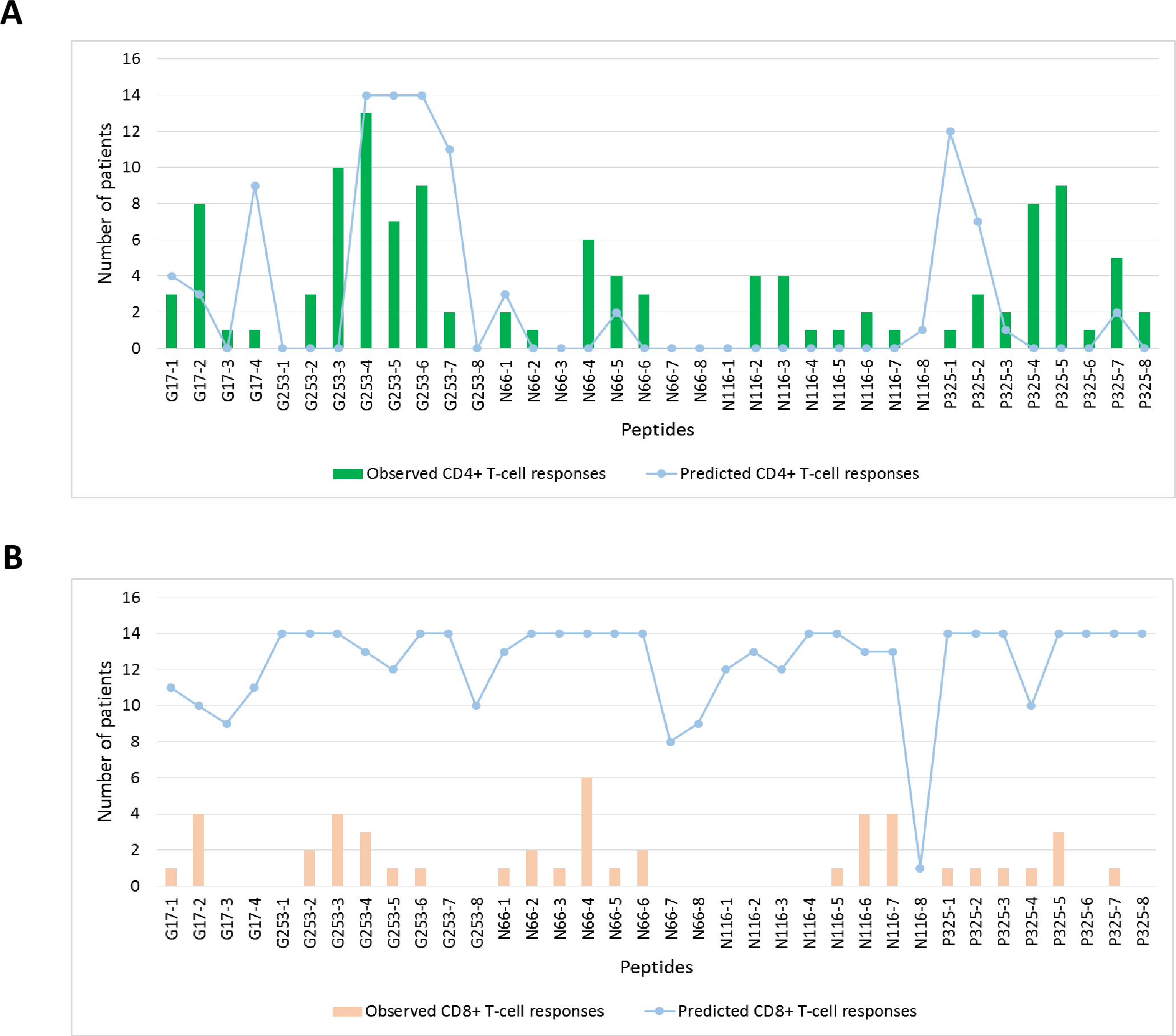

Supplement: S4 Fig — (A) CD4+ T-cell responses according to NetMHCIIpan 3.2 HLA-DRB1-binding predicted 15-mer peptides (blue line) or observed after 7-day ICS (green bars) for the 14 patients tested at W16. (B) CD8+ T-cell responses according to NetMHCpan 4.0 HLA-A/B/C-binding predicted 15-mer peptides (blue line) or observed after 7-day ICS (orange bars) for the 14 patients tested at W16. (TIF) [file ppat.1008011.s004.tif]

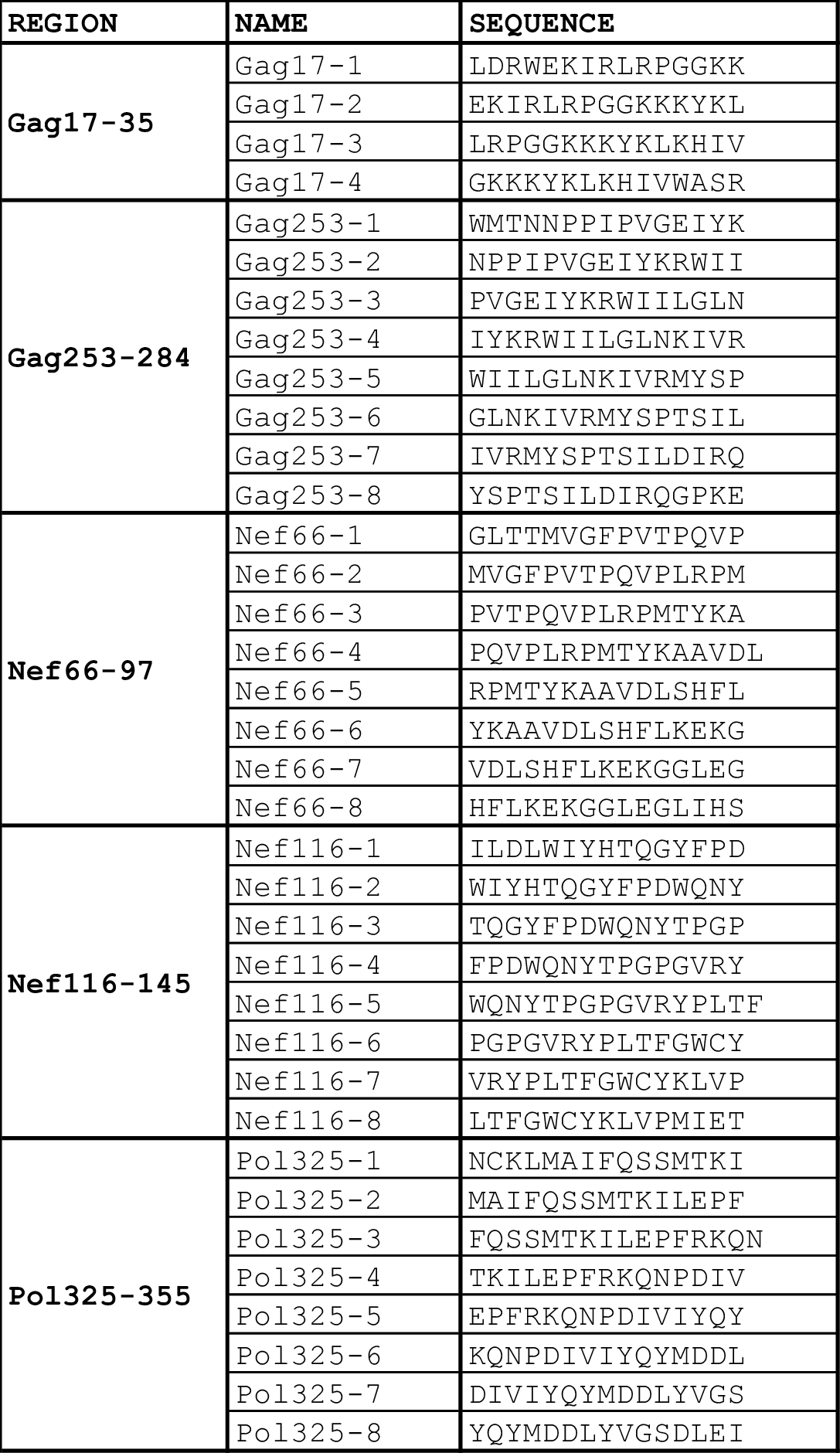

Supplement: S1 Table — (TIF) [file ppat.1008011.s005.tif]

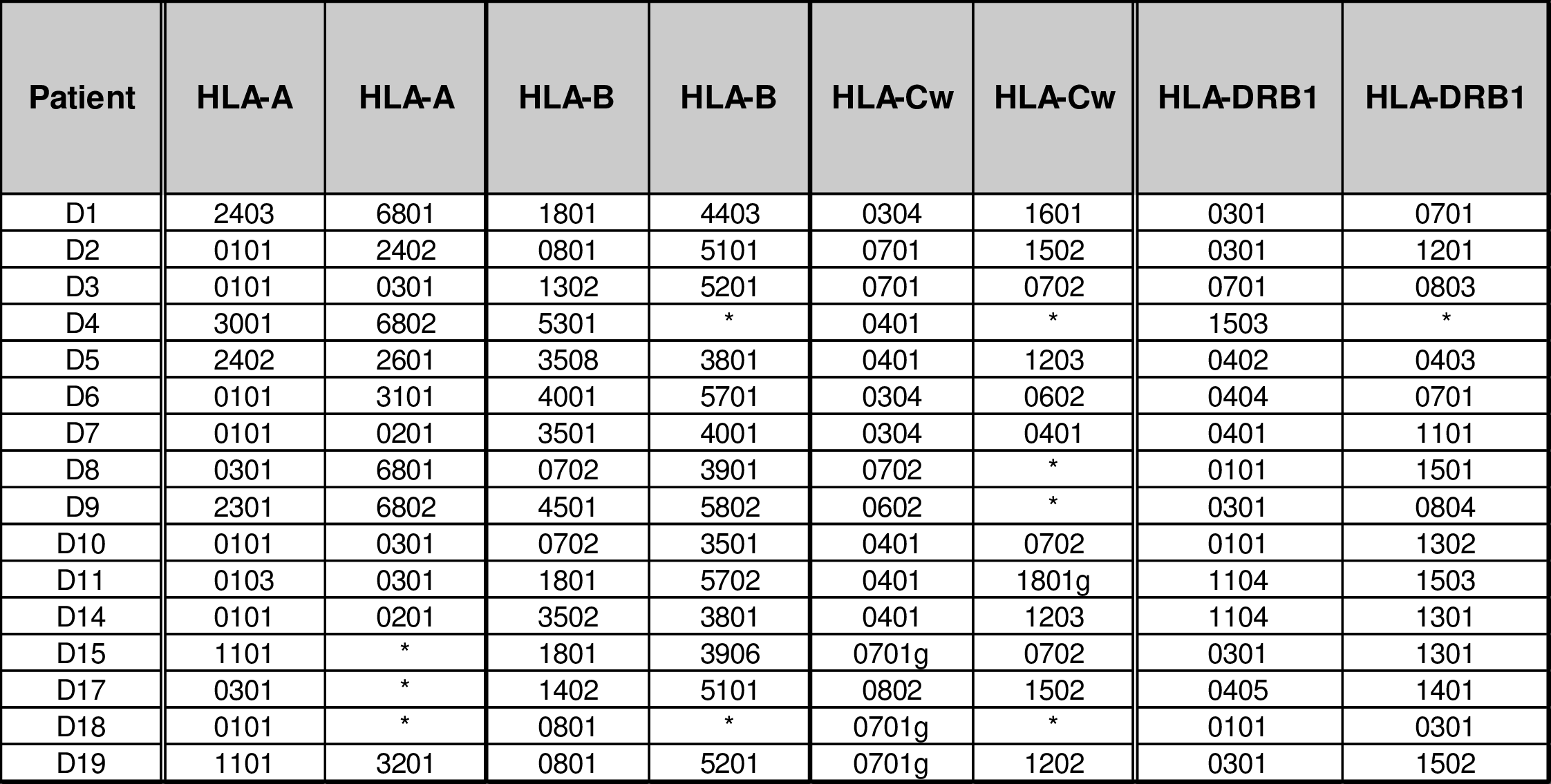

Supplement: S4 Table — (TIF) [file ppat.1008011.s008.tif]
